# The yield of common buckwheat (*Fagopyrum esculentum* Moench) depends on the genotype but not on the Pin-to-Thrum ratio

**DOI:** 10.1038/s41598-023-43059-0

**Published:** 2023-09-25

**Authors:** Agnieszka Płażek, Przemysław Kopeć, Michał Dziurka, Aneta Słomka

**Affiliations:** 1 Department of Breeding, Physiology of Plants and Seed Science, Faculty of Agriculture and Economics, University of Agriculture of Krakow, Podłużna 3, 30-239 Kraków, Poland; 2 The Franciszek Górski Institute of Plant Physiology, Polish Academy of Sciences, Niezapominajek 21, 30-239 Kraków, Poland; 3 Department of Plant Cytology and Embryology, Institute of Botany, Faculty of Biology, Jagiellonian University in Kraków, Gronostajowa 9, 30–387 Kraków, Poland

**Keywords:** Biochemistry, Cell biology, Developmental biology, Plant sciences

## Abstract

Common buckwheat has a complicated flowering biology. It is characterized by a strong self-incompatibility resulting from heterostyly, i.e. the occurrence of two types of flowers: Pin and Thrum, differing in the length of pistils and stamens. Fertilization occurs only as a result of cross-pollination between these morphs. Suspicions exist that the disturbed ratio between plants producing Pin and Thrum flowers (with the latter type generating more seeds) causes low seed yield. The aim of the study was to analyze: (1) the ratio between plants with Pin and Thrum morphs, (2) flower and seed production, as well as abortion of flowers, (3) the composition of nectar collected at an early flowering stage and during full flowering. The study was performed under semi-controlled and field conditions on six Polish accessions. The results indicated that under semi-controlled conditions the Pin-to-Thrum ratio was indeed disproportionate; such a phenomenon is called anisoplethy. In the field, however, the Pin-to-Thrum ratio was well-balanced (isoplethy). The plants with both morphs aborted a similar percentage of flowers and produced a comparable number of empty seeds. The number of flowers, their abortion, and ripe seed production were independent of flower type, however, they were genotypically controlled. A strong correlation between the number of flowers produced by a plant, flower abortion and the number of empty seeds was found. The percentage of aborted flowers correlated positively with the weight of ripe seeds. Nectar composition was similar for all buckwheat genotypes, but we found some differences in the amount of individual sugars depending on the blooming stage. In the majority of accessions, the nectar produced at the early blooming stage was characterized by a greater mass and volume, and contained more individual sugars than at the full-flowering stage.

## Introduction

Common buckwheat (*Fagopyrum esculentum* Moench) belongs to the *Polygonaceae* family, but it is classified as a pseudo-cereal crop. The plant is cultivated mainly for food due to the beneficial chemical composition of its seeds. The seeds have a very high content of starch, minerals, vitamins, rutin, antioxidants, and dietary fiber, as well as an unique amino acid composition (they are especially rich in lysine and arginine), and are gluten-free^1–6^. Additionally, buckwheat flour is richer in proteins than rice, wheat, sorghum, and maize^7^. Thanks to its high antioxidant content (e.g. phenolic compounds and rutin), this species is also recognized for its medicinal qualities^8–10^. Buckwheat seeds are mainly processed into groats and flour, while straw is used as fodder, and buckwheat nectar is a source of valuable honey^1^. In 2020, the global area of buckwheat cultivation was estimated to be 1 million 856 thousand hectares (ha) giving over 1 million 810 thousand tones yield^11^.

Despite its numerous beneficial properties, the considerable agricultural potential of common buckwheat has not yet been fully appreciated. This has presumably happened due to unstable and low seed yields caused by difficult flowering biology, female sterility, sensitivity to unfavorable environmental conditions and pre-harvest sprouting^1,3,12,13^. The low yield of buckwheat seeds is also attributed to a high degree of abnormalities that occur during embryological development and are conditioned by the genetic background^14^. Heterostyly present in this species results in its self-incompatibility. Plants produce Pin flowers with short stamens and long pistils or Thrum flowers with long stamens and short pistils. A single plant forms only one type of flowers. Fertilization occurs only as a result of cross-pollination between these two flower forms, so the seed yield depends on a well-balanced proportion between plants with Pin flowers and those with Thrum ones. Thanks to the action of a supergene consisting of 5 genes, the expected value of Pin:Thrum is 1:1 and is conserved from generation to generation. Flowers can be pollinated for only 1 day^15^. Some authors explained that the disturbed ratio of the Pin and Thrum morphs may be one of the reasons for the low yield of common buckwheat seeds^16–18^. The equilibrium of this ratio is called isoplethy, while deviations are called anisoplethy^19,20^. Buckwheat is characterized by indeterminate flowering and blooms throughout the growing season, which results in strong competition for assimilates between already set seeds and flowers still being produced^21,22^. One plant during the vegetative season can produce more than two thousands of flowers, but only 10% are fertilized and produce seeds^14,22,23^. Common buckwheat demonstrates a highly significant percentage of flower and embryo abortion which can reach between 50 and 70%^12,14^. Our previous studies indicated that the higher the flower production, the higher the percentage of flower and embryo abortion. However, the degree of aborted embryos depends on the plant genotype^14,22^. We stated that common buckwheat plants limit the number of seeds they can fill with assimilates, and increasing the number of flowers does not increase the seed number per se, but it can still raise their mass^22^. After removing 50% of inflorescences, Halbrecq et al.^24^ observed a 10–15% increase in seed set.

Pollination of common buckwheat depends on nectar productivity by flowers, their blooming stages, weather conditions, sowing time, and genetic background of the cultivar^25^. Nectar production is sensitive to environmental stresses, such as light deficiency, water stress and low temperature^3^. The nectar of buckwheat is attractive to pollinators and its production correlates with seed yield. The primary pollinators of common buckwheat are honeybees^12^. The nectar of common buckwheat consists mainly of glucose, fructose, and sucrose^1^. According to Cawoy et al.^1^, nectar mass and composition depend on flower type and plant age: Pin flowers produce less nectar and are less commonly visited by honeybees than Thrum flowers. The first flowers in anthesis produce more seeds than flowers developed later^23^. However, in other studies, the same group reported that these flower morphs produced nectar containing comparable total sugar content. According to Taylor and Obendorf^26^ and Halbrecq et al.^24^, the production of nectar and seed setting depend on the position of the flowers in a raceme, which is related to their access to assimilates.

In our previous studies carried out in a glasshouse and open foil tunnel, we observed a highly distorted proportion between Pin and Thrum flowers^14^. We reported that four genotypes of common buckwheat showed drastically lower number of plants with Pin flowers than with Thrum morphs. Moreover, Pin flowers produced less seeds. In contrast to our previous results and to results present in the literature on the subject, we hypothesize that Pin-Thrum flowers ratio in filed conditions is balanced and seed production is genotype-specific and independent of flower morph and nectar productivity.

The experiments conducted by our team aimed to analyze: (1) the ratio between Pin and Thrum morphs, (2) occurrence of the abortion of flowers, (3) flower and seed production, (4) the composition of nectar collected from fully open flowers ready for fertilization in two time points: at the beginning and full flowering stages.

## Results

### Pin-to-Thrum ratios in open foil tunnel and field experiments

The number of plants producing Pin and Thrum flowers significantly differed for each studied accession in open foil tunnel experiment (Table 1). There was no stable proportion between both morphs. The most disturbed Pin-to-Thrum ratio (1:11.5) was recorded for the PA13 line. The highest number of Pin flowers was produced by the PA15 line and the Pin-to-Thrum ratio in this case was 1:1.5. This ratio was similar for the ‘Panda’ cv. (1:4.6) and the lines PA14 and PA16 (1:5.3 and 1:4.5, respectively).

**Table 1:** Proportion between plants producing Pin (long pistil and short stamens) and Thrum (short pistil and long stamens) flowers of six common buckwheat genotypes in open foil conditions.

Genotype	Number of plants with Pin flowers	Number of plants with Thrum flowers	Pin:Thrum ratio
PA13	8	92	1:11.5
PA14	14	86	1:5.3
PA15	39	61	1:1.5
PA16	18	82	1:4.5
‘Kora’	31	69	1:2.3
‘Panda’	17	83	1:4.9

The 3-year experiment was performed on 100 plants of each genotype each year. Presented data are means (n = 3).

In field experiment, the ratio of plants with Pin flowers to plants with Thrum flower morphs was similar between all studied genotypes (Table 2). The number of plants producing Thrum flowers was slightly, but insignificantly higher than of those with Pin flowers, except for the PA16 line. These results show that analysis of a greater population of plants in the field conditions proves well balanced Pin-to-Thrum ratio when compared to the pot cultivation.

**Table 2:** The number of plants with Pin (long pistil and short stamens) and Thrum (short pistil and long stamens) flowers of six common buckwheat genotypes cultivated in the field conditions.

Genotype	Number of plants with Pin flowers	Number of plants with Thrum flowers	Pin:Thrum ratio
PA13	97	103	1:1.06
PA14	95	105	1:1.05
PA15	99	101	1:1.02
PA16	97	93	1.04:1
‘Kora’	95	95	1:1
‘Panda’	95	105	1:1.05

The 3-year experiment was performed on 200 plants of each genotype each year. Data are presented as means (n = 3).

We also found a significant correlation between the number of flowers and the percentage of flower abortion, number of empty seeds, and MTS (mass of thousand seeds) (Table 3). A strong correlation between the number of flowers and their abortion indicated that the more flowers were produced, the more were rejected. Moreover, the correlation between the number of flowers and empty seeds suggested that empty seeds were formed as an effect of aborting the embryos. The number of flowers, however, did not correlate with the number of ripe seeds and their weight calculated per plant.

**Table 3:** Correlation (Pearson coefficient; P < 0.05) between the number of flowers and their abortion, the number of empty and ripe seeds, weight of seeds (all parameters were calculated per plant), and mass of thousand seeds (MTS) obtained in the open foil tunnel.

Parameter	Number of flowers
Percentage of abortion	0.927
Number of empty seeds	0.656
Number of ripe seeds	ns
Seed mass	ns
MTS	0.592

*ns* non-significant.

### Sugar composition in nectar

Our preliminary analyses showed no differences in the composition of nectar produced by Pin and Thrum flowers, so we decided to analyze quantitative and qualitative characteristics of nectar regardless of the flower type. The nectar composition of plants grown in an open tunnel was studied. The controlled conditions of the experiment made it possible to limit the influence of environmental conditions on the quantity and quality of nectar, including rainfall and water deficit in the soil.

The analysis of variance (two-way ANOVA) showed a significant influence of genotype and flowering time, as well as the interaction of these factors on the nectar mass and volume, and individual sugar content, the sum of sugars, and fructans (Suppl. Table 1).

The nectar of common buckwheat was mainly composed of fructose, glucose and sucrose; maltose, fructans, glycerol, and inositol were also detected (Table 4). In general, we found significant differences in all studied parameters of the nectar composition from all the accessions. The nectar composition also differed between both flowering time points.

**Table 4:** Nectar composition from flowers of six genotypes of common buckwheat at two flowering stages: at the beginning of the flowering (I) and in the phase of full flowering (II).

Parameter	PA13		PA14		PA15		PA16		‘Kora’		‘Panda’	
	I	II	I	II	I	II	I	II	I	II	I	II
Nectar mass [mg/50 flowers]	88.82b	64.15e	76.14c	50.90g	65.08e	57.27f	56.45f	55.10f	88.15b	71.43d	128.18a	70.41d
Nectar volume [μl/50 lowers]	85.27b	61.78e	72.87cd	49.07g	63.22e	56.02f	54.90f	54.04f	84.60b	68.00d	122.20a	67.51d
Inositol [mg/cm^3^]	1.042a	1.031a	1.046a	1.037a	0.562b	0.121f	0.308c	0.146ef	0.119f	0.245d	0.153e	0.188e
Glycerol [mg/cm^3^]	0.261d	0.252d	0.277d	0.234d	1.061b	1.063b	1.301a	1.223a	1.090b	1.132ab	0.856c	0.949bc
Glucose [mg/cm^3^]	1.20g	1.72f	1.19g	1.76f	10.10a	6.03d	5.72de	5.07e	7.50b	6.44c	5.79d	6.75c
Fructose [mg/cm^3^]	16.24b	12.39d	23.26a	15.29bc	9.46e	6.32g	7.50f	7.40f	14.24c	12.12d	11.18de	11.53de
Sucrose [mg/cm^3^]	26.64b	21.31c	35.09a	24.01bc	1.88e	0.96f	0.46f	1.65e	2.87d	2.54d	3.08d	3.00d
Maltose [mg/cm^3^]	1.297cd	1.379c	3.525a	2.446b	0.921d	0.883d	0.791e	0.874de	0.202f	0.170g	0.269f	0.273f
Kestose [mg/cm^3^]	0.572d	0.602d	0.593d	0.644d	3.642b	2.291c	3.170bc	2.323c	4.542a	4.390a	4.851a	3.322b
Nystose [mg/cm^3^]	0.591b	0.606b	0.704a	0.451c	0.347d	0.013j	0.105h	0.059i	0.281e	0.174f	0.321d	0.156fg
Sum of sugars [mg/cm^3^]	50.78b	42.35c	69.70a	47.01bc	26.37e	15.53f	17.74f	18.34f	30.90d	28.00de	26.82e	30.36d
Sum of fructans [mg/cm^3^]	1.163e	1.208e	1.297e	1.095e	3.989bc	2.304d	3.275c	2.382d	4.823ab	4.564b	5.171a	3.478c
Percent of hexoses in total sum of sugars	34	33	35	36	74	80	75	68	70	66	63	60

Means (n = 9) marked in the lines with the same letter did not differ significantly (Duncan’s multiple-range test; P < 0.05).

#### Mass and volume of nectar

The highest nectar mass and volume were noted at the beginning of flowering in the ‘Panda’ cv. flowers, while the lowest ones were recorded in the flowers of PA14 line during the phase of full flowering (Table 4). Nectar mass and volume were greater in the first phase of flowering than in the later phase in all accessions except the PA16 line.

#### Inositol and glycerol

Inositol and glycerol contents were low and similar between both flowering terms in almost all accessions except for the PA15 and PA16 lines (Table 4). The nectar of the PA13 and PA14 flowers contained more inositol and less glycerol than the nectar of the other plant genotypes. In this case, there was a negative relationship between the contents of both sugars: the higher the content of inositol, the lower the content of glycerol.

#### Hexoses and sucrose

Considerable differences were found in the amount of glucose for all studied genotypes (Table 4). The nectar of PA15 plants contained the highest amount of glucose. For this line, it was also greater in the first than in the second flowering term. The lowest amount of glucose was found in the flowers of PA13 and PA14 lines. In contrast to other accessions, the nectar of PA13 and PA14 lines contained a drastically higher level of fructose than glucose. The lowest amount of this hexose was noted in the nectar of PA15 in the second flowering phase, and in PA16 at both blooming stages. Except for PA16 and cv. ‘Panda’, fructose concentration in nectar decreased during the second phase of flowering. The highest percent of hexoses in relation to total sugar amount was detected in the line PA15 (70% and 80% in the two blooming phases, respectively), while the lowest total sugar amount was present in the nectar of the lines PA13 and PA14. No significant differences in hexose amounts were found between the beginning of blooming and the full flowering stage. The nectar of PA13 and PA14 showed repeatedly greater amount of sucrose at both flowering stages than in the other accessions. The lowest amount of sucrose was noted in the nectar of PA15 and PA16 at both blooming stages. In PA13 and PA14 lines, as well as in the line PA15, sucrose concentration in the second flowering phase was lower than at the beginning of blooming.

#### Maltose, fructans, sum of sugars

The nectar of PA13 and PA14 contained a significantly greater amount of maltose than the nectar of the other genotypes (Table 4). In the lines PA13, PA14, and PA15, the concentration of maltose was higher at the beginning of flowering than at full bloom. The nectars of all studied genotypes also contained fructans, such as kestose and nystose. The concentrations of kestose and nystose were similar between the lines PA13 and PA14. In the other accessions, kestose concentration was considerably higher than nystose level. The PA15 and PA16 lines, and cv. ‘Panda’, contained higher fructan level at the beginning of the flowering stage than at the second flowering phase. In the other plant genotypes, the amount of fructans was similar during both phases of blooming. In the flowers of PA13, PA14, and PA15, the total amount of sugars in the nectar was greater at the beginning of flowering.

To investigate whether the quantity and quality of the nectar may affect the buckwheat yield, we determined the plant yield parameters for each of the examined genotypes (Table 5). The lines PA13 and PA16 produced the most seeds. However, the PA15 line formed fewer of seeds, but they were the heaviest. No correlation between the amount of individual sugars in nectar and seed yield was found, however, glucose amount in the nectar correlated negatively with the number of flowers produced (r = – 0.898; P < 0.05) and the percentage of flowers and embryos aborted (r = – 0.982; P < 0.05). In turn, abortion of flowers and embryos correlated negatively with nectar mass (r = – 0.897; P < 0.05) and its volume (r = – 0.891; P < 0.05). Moreover, there was a correlation between the nectar mass and the seed weight (r = 0.867, P < 0.05).

**Table 5:** Yield parameters of six genotypes of common buckwheat grown in an open foil tunnel: number of ripe seeds and their mass produced by plant, and a mass of thousand seeds (MTS).

Line/cultivar	No. ripe seeds/plant	Seed mass/plant [g]	MTS [g]
PA13	126.5a	3.29 ± b	26.11 ± b
PA14	91.9cd	2.64 ± d	29.03 ± a
PA15	93.9cd	3.69 ± a	29.99 ± a
PA16	116.5ab	2.64 ± d	28.21 ± a
‘Kora’	104.6bc	3.06 ± c	25.91 ± b
‘Panda’	87.6d	2.53 ± e	28.88 ± a

Means (n = 20) marked with the same letter do not differ significantly (multiple range Duncan’s test; P < 0.05).

### Flower and seed production, and flower abortion in filed conditions

A significant impact of genotype on all the studied parameters with the exception of the percentage of flower abortion was shown (Suppl. Table 2). Flower morph decided the number of flowers the plant produced, the number of ripe seeds, their weight, and MTS. The interaction between these factors was significant only in the case of flower number and seed weight.

The PA13, PA14, and PA16 accessions produced more Pin flowers than Thrum ones when calculated per plant (Table 6). Plants of line PA15 and cvs. ‘Kora’ and ‘Panda’ produced the same number of both flower morphs, while line PA15 showed the highest number of both types of flowers.

**Table 6:** The number of Thrum and Pin flowers, number of empty seeds, and percentage of empty seeds in relation to all seeds produced by both flower morphs, ripe seeds produced by each type of flower, ripe seed mass – all parameters calculated per plant, and the mass of a thousand seeds (MTS) of six common buckwheat genotypes cultivated in the field conditions in four replicates each year.

Line/cultivar	Type of flower	Number of flowers/plant	Number of empty seeds/plant	Percentage of empty seeds [%]	Number of ripe seeds/plant	Abortion of flowers and embryos [%]	Seed mass/plant [g]	MTS [g]
PA13	Thrum	805d	106.5bc	32.0bc	226.0e	71.9a	6.18fg	23.48e
	Pin	912bc	105.6bc	28.3c	267.5b	70.7a	7.51bc	28.07c
PA14	Thrum	926bc	93.9c	29.7c	222.6e	76.0a	6.66ef	29.96b
	Pin	1007a	117.3bc	29.1c	286.0a	71.6a	8.55a	29.91b
PA15	Thrum	1027a	127.3b	34.2ab	244.8cd	76.2a	7.75b	31.63a
	Pin	1031a	167.7a	39.1a	261.2bc	74.7a	8.39a	32.09a
PA16	Thrum	883c	98.7c	29.5c	236.4de	73.2a	7.14cd	28.12c
	Pin	937b	114.2b	29.0c	279.3ab	70.2a	6.72de	28.29c
‘Kora’	Thrum	910bc	97.7c	27.0cd	263.8b	71.0a	7.17cd	27.18d
	Pin	936b	96.0c	25.8d	275.6ab	70.6a	7.83b	28.41c
‘Panda’	Thrum	787d	118.5bc	35.2ab	218.0e	72.3a	6.11g	28.11cd
	Pin	800d	109.3bc	33.1b	220.6e	72.4a	6.37efg	28.83c

Means (n = 12) (25 plants per plot comprised one replicate) marked with the same letter did not differ significantly (Duncan’s multiple-range test; P < 0.05).

The same number of empty seeds was obtained in the case of PA13 and cv. ‘Kora’ from both flower morphs (Table 6). In PA14, PA16, and PA15 accessions, Pin flowers produced a higher number of empty seeds than Thrum flowers; however, only in the case of PA15 this difference was significant. Pin flowers of the line PA15 produced the greatest amount of empty seeds among all studied accessions and flower morphs. The highest percentage of empty seeds in relation to all produced seeds was calculated for both morphs of PA15 line, and this effect was related to the highest number of both flower types of this line. No differences between the percentage of empty seeds obtained from Pin and Thrum flowers were found in any accession.

In the lines PA13, PA14, and PA16 more ripe seeds were produced by Pin flowers than Thrum morphs. Both cultivars (i.e. ‘Kora’ and ‘Panda’) and line PA15 produced the same ripe seed yield for each flower type. However, the highest ripe seed number was obtained from Pin flowers of lines PA14, PA16, and cv. ‘Kora’.

The percentage of abortion of flowers and embryos was similar for all the types of flowers and accessions (Table 6). In the case of most genotypes (except for the line PA16 and cv. ‘Panda’), the seeds obtained from Pin flowers demonstrated greater weight. The greatest weight of seeds was obtained from Pin flowers of PA14 and PA15 plants, while the smallest seeds were produced by both types of flowers of cv. ‘Panda’ and Thrum flowers of PA13 line.

Small but significant differences were found between the mass of thousand seeds of individual accessions (Table 6). The PA15 line demonstrated the highest MTS for both flower types, while the lowest MTS was noted for the Thrum morphs of line PA13.

A significant correlation was found between the number of flowers and ripe seeds, their weight, and MTS (Table 7). In contrast with the results from the pot experiment, no correlation between the number of flowers and percentage of abortion, and number of empty seeds was shown. In both cases, the abortion of flowers correlated with MTS (r = 0.592 and 0.727; P < 0.05).

**Table 7:** Correlation (Pearson coefficient; P < 0.05) between the number of flowers and their abortion, number of empty and ripe seeds, seed weight (all parameters calculated per plant), and the mass of a thousand seeds (MTS) obtained in the field conditions.

Parameters	Number of flowers
Percentage of abortion	ns
Number of empty seeds	ns
Number of ripe seeds	0.662
Seed weight	0.874
MTS	0.727

*ns* non-significant.

## Discussion

### Pin and Thrum morph ratio

In our previous investigation, in four common buckwheat lines and cultivars (PA13, PA14, ‘Kora’ and ‘Panda’), we observed a disturbed ratio of the Pin and Thrum morphs of all accessions^14^. In the present study, we added two new lines, PA15 and PA16.The results obtained in the experiment carried out under semi-controlled conditions (i.e. in an open foil tunnel) showed disturbed proportion between Pin and Thrum morphs of all studied accessions. These results confirm our previous findings for PA13, PA14, and cvs. ‘Kora’ and ‘Panda’^14^. In both tunnel experiments, line PA13 exhibited the greatest anisoplethy, while cv. ‘Kora’ demonstrated the lowest one. However, an analogous ratio analysis performed for two hundred plants of each accession under field conditions indicated that all studied accessions were characterized by isoplethy. Quinet et al.^27^ also reported equal frequencies of Pin and Thrum morphs of common buckwheat. According to Barret and Shore^28^, anisoplethy can be observed in a small population, and it can be caused by vegetative reproduction. In both locations, where the experiments were conducted, the climatic conditions during the growing season are similar, except for the number of sunny days: in Krakow there are 151 h less than in Palikije. Although the difference seems to be significant, we do not suppose that it determines the ratio between the number of developed flowers of both morphs. However, in the future, we intend to conduct research to check the effect of different light spectrums and temperature on the formation of both types of flowers. Especially since Matsui et al.^29^ revealed that the genes for self-compatibility might show partial dominance or operate a late-acting system sensitive to environmental conditions. On the other hand, based on this study and previously published research, we suggest that the balanced proportion between Pin and Thrum plants is a population-level trait. Therefore, we recommend that the analysis of the morph ratio should be performed in a large population of plants under the conditions as close to natural ones as possible.

### Nectar production

The volume and mass of buckwheat nectar depend on the genotype, time of day (light intensity), and flower location on the plant^1,3,25^. Buckwheat blooms for long during its vegetation, so various insects often visit its flowers. Farkas and Zajácz^30^ highlighted that the production of nectar in buckwheat depends mainly on the optimal environmental conditions. The species produces definitely more nectar at temperatures not exceeding 25 °C, and with sufficient air and soil humidity. Alekseyeva and Bureyko^25^, however, reported that high sugar content of the flowers themselves affects the composition of nectar. Other researchers suggest that Thrum flowers produce more nectar than flowers of Pin morphs^23^. Later on, the authors did not confirm their previously obtained results, they concluded that although Thrum flowers had the same amount of total sugars as Pin ones, they contained higher amount of sucrose^1^. Farkas and Zajácz^30^ found that in the field the flowers of Thrum morphs were preferentially visited by pollinators who spent more time on their inflorescences as compared with flowers of Pin morphs. Płażek et al.^31^ demonstrated a positive correlation between the total amount of sugars and amino acids in the nectar and seed yield in two accessions PA15 and PA16 with biased morph ratio.

Buckwheat nectar contains mainly fructose, glucose and sucrose, with predominance of hexoses^1^. Some authors showed that hexoses account for 55–85% of all sugars under controlled conditions, and the Thrum flowers produced 30% more nectar than Pin flowers^23^. Additionally, Lee and Heimpel^32^ demonstrated that buckwheat produces the highest amounts of nectar in the early morning. In our study, the hexose content analyzed at two stages of flowering did not differ much, however, some differences were noted between the studied genotypes. The nectar of PA15 contained the highest amount of hexoses (74% and 80% of the total sum of sugars in the first and second flowering phase, respectively). In contrast, the lowest hexose content was detected in the nectar of PA13 and PA14 during both flowering phases.

We found that the composition of buckwheat nectar largely depends on the genotype and the flowering stage. All accessions produced the same compounds but in different amounts. For example, in the flowers of lines PA13 and PA14, sucrose was the main component of nectar. The nectar of lines PA15 and PA16 contained similar amounts of fructose and glucose accompanied by six times lower amount of sucrose. On the other hand, cvs. ‘Kora’ and ‘Panda’ were characterized by two times higher fructose content as compared with glucose. Additionally, sucrose content in the nectar produced by both cultivars was two to three times higher than the amount of glucose, and six to seven times higher than that of fructose. Furthermore, we determined the amounts of inositol, glycerol, maltose, and fructans (i.e. kestose and nestose) in the nectar of all accessions. It turned out that the contents of these sugars also depended on the genotype. The highest amount of total sugars was recorded in the nectar of lines PA13 and PA14, while the line PA16 was characterized by the lowest total sugar content.

We performed the nectar analysis at two flowering stages: at the beginning of flowering and in the full blooming phase, at three-week intervals. In the case of lines PA14 and PA15, the amounts of almost all nectar components were higher at the beginning of flowering than in the second phase. On the contrary, the nectar of line PA13 and cv. ‘Kora’ had more fructose and sucrose at the beginning of flowering. In all accessions (except for PA16 line), the nectar mass and volume were greater at the beginning of flowering than at the full flowering stage. Our results may confirm earlier report by Alekseyeva and Bureyko^25^ who showed that nectar productivity depends on blooming stages. During the first flowering stage plants produce more nectar than in the second phase. Cawoy et al.^23^ reported that the highest nectar amount is produced during the flowering peak. Alekseyeva and Bureyko^25^ argued that there was a strong correlation between nectar abundance and seed yield. In the current study, we only found a correlation between the nectar mass and seed weight, while the content of any of the individual sugars did not affect the seed yield. Although the statistical analysis did not show any correlation between individual sugars and seed yield, the highest content of hexoses seems to determine the highest seed yield obtained from PA15 plants. Moreover, the negative correlation between glucose amount and the number of flowers’ production as well as the percentage of flowers and embryos aborted was described. This result suggests how the overproduction of flowers depletes this sugar content. Additionally, negative correlation between abortion of flowers and embryos correlated negatively with nectar mass and its volume, which means that flowers producing more nectar were not aborted, which also contributed to better nourishment of the embryo.

### Flower productivity, abortion, and seed yield

In both experiments, the studied accessions of common buckwheat produced a different number of flowers. In the experiment performed in the open foil tunnel (semi-controlled conditions; optimally watered plants), there was a very strong correlation (r = 0.927; P < 0.05) between the number of flowers and the percentage of flower abortion. Line PA15 had the highest number of flowers, and cv. ‘Kora’ the lowest. The percent of aborted flowers was similar for lines PA13, PA14, PA15, and PA16, and it ranged between 86.7% and 91.9%. The cvs. ‘Kora’ and ‘Panda’ showed significantly lower rate of flower abortion: 67.4% and 79.1%, respectively. Cawoy et al.^15^ observed the same number of flowers on both morph plants under controlled conditions of a growth chamber. However, the authors experimented on only one cultivar ‘La Harpe’. In the present study, in contrast to the semi-controlled conditions, the percentage of flower abortion in the field was similar for all studied accessions and reached about 70%. We observed no differences in flower abortion between the Pin and Thrum morphs. In the field, the plants with Pin flower morphs of lines PA13, PA14, and PA16 produced more flowers than those with Thrum morphs. In the other accessions, flower production was similar in both Pin and Thrum plants. These results show that flower abortion does not depend on flower morph but is related to genotype.

A low percentage of seed setting in relation to produced flowers is a characteristic feature of common buckwheat. According to Jacquemart et al.^12^ and Farooq et al.^3^, internal defects are responsible for seed production failure, namely male or female sterility or embryo abortion. Our previous studies^14,33^ documented very high pollen viability (99%) and germination of all studied Polish accessions of common buckwheat, so the main reason for the lack of fertilization is the high percentage of degenerated embryo sacs. The rate of embryo abortion depends on the genotype. Among the studied accessions, we observed significant differences in the percentage of incorrectly developed ovules, both under control conditions and under thermal and nutrient stresses^14,22,33,34^.

Non-pollinated flowers are usually rejected by plant, but Taylor and Obendorf^26^ reported that 76–91% of pollinated flowers showed no signs of fertilization. Furthermore, Halbrecq et al.^24^ and Cawoy et al.^35^ found that seed production is not possible from all flowers. Still, this ability depends on the flower localization on the plant and the first flowers in anthesis are more likely to produce seeds than the later ones. This opinion was also shared by Björkman^17^ as well as by Taylor and Obendorf^26^.

In the presented experiments, we analyzed flower productivity; empty and ripe seed number calculated per plant. Our earlier embryological analyses showed that even if fertilization is completed, the developing embryo often dies due to starvation, resulting from insufficient assimilate supply to the developing seed^34^. In contrast to our opinion, Halbrecq et al.^24^ and Cawoy et al.^35^ suggested that embryo abortion in common buckwheat is fixed by an internal mechanism and it does not depend on insufficient nutrient supply from leaves. Against this suggestion, but in line with our opinion, Inoue and Hagiwara^21^ believe that low flower fertilization and seed set is caused by intense competition for nutrients between the donor leaves and flower acceptors. Taking this fact into account, we treated the empty seeds as a sign of embryo abortion. In the experiment performed in the open foil tunnel, the percentage of empty seeds was in accordance with the percentage of flower abortion evaluated based on the number of flowers and ripe seeds: the lower percent of empty seeds was found for cvs. ‘Kora’ and ‘Panda’ characterized by lower degree of flower abortion as compared with other accessions. Under semi-controlled conditions, a strong correlation between the number of flowers and the number of empty seeds was shown (r = 0.656; P < 0.05). Interestingly, we found no correlation between flower production and seed number.

In contrast with the pot conditions, the number of flowers in the field correlated with the number of ripe seeds, their weight, and MTS, whereas the abortion rate correlated only with MTS. The latter result means that the higher the flower abortion, the greater mass of the seeds. The lowest percentage of empty seeds per total seed yield was found  for cv. ’Kora’, while the highest percentage of empty seeds was recorded for the line PA15, with the highest number of flowers produced per plant. It should be highlighted that plants with Pin flowers had the same percentage of empty seeds as those with Thrum morphs.

In our earlier study performed in the open foil tunnel^14^, Pin morphs of cvs. ‘Kora’ and ‘Panda’ produced more seeds than Thrum ones. However, in both lines PA13 and PA15 Thrum morphs produced more seeds than Pin plants. In the current field experiment, both flower morphs of cvs. ‘Kora’ and ‘Panda’ produced similar number of seeds, while in the other accessions, more seeds were obtained from Pin flowers than from Thrum ones. This finding contradicts the report of Farkas and Zajácz^30^ who showed that pollinators prefer Thrum flowers, which would result in a higher yield of seeds from this type of flower as compared with Pin morphs.

Under semi-controlled conditions, we found the highest number of ripe seeds in the lines PA13 and PA16, while the highest seed weight per plant was determined for the line PA15. In the field experiment, cv. ‘Kora’ demonstrated the highest number of ripe seeds, but the line PA15 was characterized by the highest seed weight. Based on the mass of one thousand seeds, it can be assumed that cv. ‘Kora’ produced the lightest seeds, while PA15 line developed the heaviest seeds. The MTS was correlated with the weight of seeds collected from a single plant. The results obtained in the control conditions are not always consistent with those from the field. Based on the findings of our study, it can be concluded that the PA15 line had the highest seed yield in both experiments, especially in terms of seed weight. It produced the highest number of flowers and empty seeds as well as seeds of the greatest weight in the field conditions. Values of these parameters only partly lined up with those obtained from the foil tunnel experiment. This included the percentage of empty seeds, flower abortion, and seed weight per yield obtained from a single plant.

One of the aims of our study was to compare the results obtained in the foil tunnel with those obtained in the field. Therefore, we can assume that specific population-level traits can only be assessed under conditions as close to natural as possible and, importantly, on a larger population of plants and cultivars than can be used under greenhouse or tunnel conditions.

### Conclusions

Under the field conditions, the ratio of common buckwheat plants with Pin flowers to plants with Thrum morphs indicates isoplethy, and it is a population-level trait. We proved that plants with different flower morphs produce different flower numbers depending on their genotype. Percentage of flower and embryo abortion is a characteristic trait of each genotype. Also seed yield depends on the genotype of common buckwheat, but not on flower morph.

Number of flowers produced by plant correlates with flower abortion and number of empty seeds, and flowers abortion correlates positively with the weight of ripe seeds. Nectar composition is the same for all buckwheat genotypes, but it may differ in the amount of individual sugars as well as the mass and volume. In turn, nectar produced at the early blooming stage is characterized by a greater mass and volume in most genotypes of common buckwheat. It contains more individual sugars than at the full flowering stage.

### Methods

The study consisted of two independent experiments: in the open foil tunnel under semi-controlled conditions, and in the field. Both experiments were carried out for 3 years. The data reported in the tables show the mean results obtained within three years. Thus, the number of replicates for each analysis performed every year was multiplied by three.

### Plant material and cultivation

The study was performed on six Polish accessions of common buckwheat: two cultivars (i.e. ‘Kora’ and ‘Panda’), and four lines: PA13, PA14, PA15, and PA16. The seeds were obtained from the Plant Breeding and Production Center in Palikije (Poland), a branch of Malopolska Plant Breeding (Polanowice, Poland). The collection of plant material and the performance of experimental research on such plants complied with the national guidelines of Poland. The plants were cultivated in the open foil tunnel of the University of Agriculture in Kraków (Poland), located at the latitude 50° 04′ 10″ N and longitude 19° 50′ 44″ E, and in the experimental field of the Plant Breeding and Production Center in Palikije (51° 13′ 55″ N; 22° 18′ 33″ E). In Kraków, during the growing season from May to September, the average temperature is 20 °C, the total rainfall is 480 mm, and the number of sunny hours is 1449. In Palikije, in this season, the average temperature is 19 °C, the total rainfall is 380 mm, and the number of sunshine hours is 1600 (www.climate-data.org).

#### Open foil tunnel

Buckwheat seeds were sown into pots (20 × 20 × 25 cm, five plants per pot), filled with commercial soil substrate mixed 1:1 (v:v) with perlite. The seeds were sown in 25 pots for each accession. The experiment was established in mid-May in an open foil tunnel, which enabled us to control the substrate humidity, and to ensure free access for pollinators. The plants were fertilized once a week with a multi-component fertilizer Florovit (GRUPA INCO S.A., Warsaw, Poland). The ripe seeds were harvested at the end of August.

#### Field conditions

The experiment was performed on loess soil, and winter triticale was used as the forecrop. Due to the high soil content of nutrients, no mineral fertilizer was used. The seeds were sown on May 20, and the plants were cultivated until August 30 each year, until the harvest of the ripe seeds.

The experiment was set up on four plots (one plot 5 m^2^) for each genotype. The seeds were sown manually at a distance of 20 × 10 cm from each other, 3–4 seeds per point. After the emergence, the seedlings were interrupted, leaving one plant per point, which resulted in 250 plants per plot.

### Methods

#### Flower and seed production, abortion of flowers

In open foil tunnel, during the flowering phase, the ratio of plants with Thrum flowers to those with Pin ones was analyzed for 100 plants of each genotype each year. The flower number was counted at the end of the full flowering phase for 20 plants of each accession (5 plants per pot, 4 replicates each year). Then, at the end of the growing season, when the most of the seeds were ripe, the number of empty and ripe seeds, their mass, and the mass of a thousand seeds (MTS) were analyzed for 20 plants of each genotype (in four replicates each year).

In field conditions, the ratio of plants producing Thrum flowers to those with Pin flowers was analyzed during the full flowering phase each year for 200 plants of each line and cultivar.

The number of the flower morphs per plant, the number of empty and ripe seeds, the mass of ripe seeds per plant, and the mass of the thousand seeds produced by each flower morph were counted for 100 plants of each line and cultivar in four replicates each year (25 plants from one plot was one replicate). Then, based on the number of ripe seeds and flowers calculated per plant, the percentage of flower abortion was calculated according to the following formula:

$$\left( {{1} - \,number \, of \, ripe \, seeds/number \, of \, flowers} \right) \, \times { 1}00\% .$$

The empty seeds were recognized as dead embryos, which, according to our previous research^17^, died as a result of starvation (i.e. the lack of a sufficient amount of assimilates delivered to the developing embryo).

#### Sugar composition in nectar

The sugar composition in nectar was assessed using the method reported by Hura et al.^36^. The assessment took place over the two time points: at the beginning of flowering and in the phase of full flowering. The nectar was obtained from 50 open flowers per plant, three plants per genotype. The flowers were collected in the morning, between 8.00 and 10.00 a.m. We did not examine Pin and Thrum plants separately as our unpublished results had showed that there were no differences between both flowers morphs. The samples were extracted with ultra-pure water by shaking for 15 min at 30 Hz (MM 400, Retsch, Haan, Germany) and centrifuged for 5 min at 21,000×*g* (Universal 32R, Hettich, Germany). Then, the supernatant was collected, diluted with acetonitrile 1:1 (v/v), filtered (0.22 μm nylon mesh, Costar Spin-X, Corning, USA). The filtrate was then subjected to analysis using high-performance liquid chromatography (HPLC) with the Agilent 1200 binary system (Agilent, Wolbrum, Germany) coupled with ESA Coulochem II electrochemical detector (ESA, Chelmsford, MA, USA). An RCX-10, 7 μm, 250 × 4.1 mm column (Hamilton, Reno, NV, USA) in a 75 mM NaOH solution gradient mode, and 500 mM sodium acetate in 75 mM NaOH solution at 1.5 cm^3^ min^–1^ were used. Pulsed amperometric detection was employed on a gold electrode. The concentrations of glycerol and sugars such as glucose, fructose, sucrose, maltose, kestose, and nystose were determined using the standards purchased from Sigma-Aldrich (Poznań, Poland). All analyses were performed in three replicates each year (on three plants per genotype).

### Statistical analysis

Analysis of variance (one- or two-way ANOVA) was performed using the STATISTICA 13 package (Statsoft, Tulsa, OK, USA). The significance of differences between means was analyzed according to the Duncan’s multiple range test at P < 0.05. Values represent means ± SE (standard error). The Pearson’s correlation coefficients were assumed significant at P < 0.05.

### Supplementary Information

Supplementary Tables.

## Data Availability

The data presented in this study are available on request from the corresponding authors.
