# Supplementary material for: The yield of common buckwheat (Fagopyrum esculentum Moench) depends on the genotype but not on the Pin-to-Thrum ratio
Source: Sci Rep. 2023 Sep 25;13:16022. doi: 10.1038/s41598-023-43059-0 (PMC10519966; doi:10.1038/s41598-023-43059-0)
Supplement: Supplementary file 1 — Supplementary Tables. [file 41598_2023_43059_MOESM1_ESM.docx]

| **Variable** | **Nectar mass** | **Nectar volume** | **Inositol** | **Glycerol** | **Glucose** | **Fructose** | **Sucrose** | **Maltose** | **Kestose** | **Nystose** | **Sum of sugars** | **Sum of fructans** |
| --- | --- | --- | --- | --- | --- | --- | --- | --- | --- | --- | --- | --- |
| Genotype | *** | *** | *** | *** | *** | *** | *** | *** | ** | ** | ** | ** |
| Stage | ** | ** | ** | ** | * | ** | *** | ** | ** | ** | ** | ** |
| G × S | ** | ** | * | * | * | ** | *** | * | * | * | * | * |

**Supplementary Table 1.** Analysis of variance (two-way ANOVA) of the effects of genotype (G), flowering stage (S), and interaction between these factors on the mass and volume of nectar, individual sugar contents, sum of sugars and fructans. Nectar composition was determined in the plants grown under an open foil tunnel; *P < 0.05; **P < 0.01; ***P < 0.001.

| **Variable** | **Number of flowers** | **Number of empty seeds** | **% of empty seeds** | **Number of ripe seeds** | **Abortion**  **of flowers and embryos** | **Seed mass** | **MTS** |
| --- | --- | --- | --- | --- | --- | --- | --- |
| Genotype | ** | * | ** | *** | ns | *** | ** |
| Flower morph | ** | ns | ns | ** | ns | ** | * |
| G × F | *** | ns | ns | ns | ns | * | ns |

**Supplementary Table 2.** Analysis of variance (two-way ANOVA) for the effects of genotype (G), flower morph (F), and interaction between these factors on the number of Thrum and Pin flowers, empty seeds, and percentage of empty seeds in relation to all seeds produced by both flower morphs, ripe seeds produced by each type of flower, ripe seed mass, and mass of a thousand seeds (MTS). *P < 0.05; **P < 0.01; ***; P < 0.001; ns – non-significant.
